# Transcriptional Profiling in a Novel Swine Model of Traumatic Brain Injury

**DOI:** 10.1089/neur.2021.0051

**Published:** 2022-04-19

**Authors:** Samuel S. Shin, Amy C. Gottschalk, Vanessa M. Mazandi, Todd J. Kilbaugh, Marco M. Hefti

**Affiliations:** ^1^ Department of Neurology, Hospital of University of Pennsylvania, Perelman School of Medicine, Children's Hospital of Philadelphia, University of Pennsylvania, Philadelphia, Pennsylvania, USA.; ^2^ College of Liberal Arts and Sciences, University of Iowa, Iowa City, Iowa, USA.; ^3^ Department of Anesthesiology and Critical Care Medicine, Children's Hospital of Philadelphia, University of Pennsylvania, Philadelphia, Pennsylvania, USA.; ^4^ Department of Pathology, University of Iowa, Iowa City, Iowa, USA.

**Keywords:** controlled cortical impact, pig, RNA, swine, traumatic brain injury

## Abstract

Transcriptomic investigations of traumatic brain injury (TBI) can give us deep insights into the pathological and compensatory processes post-injury. Thus far, transcriptomic studies in TBI have mostly used microarrays and have focused on rodent models. However, a large animal model of TBI bears a much stronger resemblance to human TBI with regard to the anatomical details, mechanics of injury, genetics, and, possibly, molecular response. Because of the advantages of a large animal TBI model, we investigated the gene expression changes between injured and uninjured sides of pig cerebral cortex after TBI. Given acute inflammation that follows after TBI and the important role that immune response plays in neuroplasticity and recovery, we hypothesized that transcriptional changes involving immune function will be upregulated. Eight female 4-week-old piglets were injured on the right hemisphere with controlled cortical impact (CCI). At 5 days after TBI, pericontusional cortex tissues from the injured side and contralateral cortical tissues were collected. After RNA extraction, library preparation and sequencing as well as gene expression changes between the ipsi- and contralateral sides were compared. There were 6642 genes that were differentially expressed between the ipsi- and contralateral sides, and 1993 genes among them had at least 3-fold differences. Differentially expressed genes were enriched for biological processes related to immune system activation, regulation of immune response, and leukocyte activation. Many of the differentially expressed genes, such as CD4, CD86, IL1A, IL23R, and IL1R1, were major regulators of immune function. This study demonstrated some of the major transcriptional changes between the pericontusional and contralateral tissue at an acute time point after TBI in pigs.

## Introduction

Traumatic brain injury (TBI) induces various pathological and compensatory biological processes, both acutely and chronically after injury.^1–3^ Among these processes, inflammation, apoptotic cascades, neural remodeling and regeneration, as well as neurodegenerative processes are widely reported.^4–8^ Developments in transcriptomic technologies, such as microarrays and RNA sequencing (RNA-seq), can provide deeper insight into the complex changes in injured tissue after the initial insult.

Most previous transcriptomic studies of TBI have utilized rodent models. Although an excellent high-throughput model for mechanistic studies, rodent TBI models are problematic for translational studies because of significantly smaller brain size, genetic differences, and immune response. The mechanics of injury differ significantly between rodents and humans.^9^ Little human data exist because of the logistic difficulties in obtaining well-characterized acute human TBI brain tissue. There is therefore an urgent need for a good human surrogate model for translational TBI studies. There is increased interest in non-canonical animal models of TBI, including swine, but the transcriptional response to TBI in swine, a critical resource for mechanistic studies, has not been systematically described.^10^ One previous study that examined transcriptional changes in response to TBI have focused on studying the effect of a drug treatment, and it did not include a control group.^11^

In the current study, we aimed to gain insight into the transcriptional changes in the pericontusional cortex using a controlled cortical impact (CCI) model of pediatric TBI. This model used 4-week-old pigs, which is equivalent to 1–2 years of human age based on the rate of brain growth and development.^12,13^ We hypothesized that various immune-mediated functions will be upregulated in the pericontusional cortex of a pig compared to the contralateral side. Previous transcriptomic studies of TBI have also mostly used gene expression microarrays.^14–17^ Whereas microarray utilizes a hybridization technique with only pre-defined transcripts, RNA-seq enables the full transcriptome to be reviewed. Thus, a greater number of differentially expressed genes can be identified compared to microarrays.^18^ Given this advantage of obtaining a more-complete analysis of the transcriptome, recent studies in TBI utilized RNA-seq instead of microarrays.^11,19–21^ In the current report, we aimed to outline the transcriptomic difference between the ipsi- and contralateral side of injury at 5 days to assess the effect of injury in a pediatric pig model of TBI.

## Methods

### Surgical preparation and injury

This study was approved by the Institutional Animal Care and Use Committee of the University of Pennsylvania (Philadelphia, PA). Eight female domestic pigs (*sus scrofa domesticus*) at 4 weeks of age underwent CCI as previously described.^22^ Briefly, pigs were pre-medicated with an intramuscular injection of ketamine (20 mg/kg) and xylazine (2 mg/kg). They then underwent induction using 4% inhaled isoflurane and intubation. Anesthesia was maintained with 1% inhaled isoflurane, and a surgical incision was made over the cranium followed by craniotomy. After exposure of the dura, a spring-loaded CCI was used to injure the brain at 4 m/s with 0.7 cm of injury depth, which is equivalent to a mild-moderate degree of injury. During the experiment, various physiological parameters were monitored and kept in close range. Mean end-tidal CO_2_ was measured at 39.7 ± 5.9 mm Hg. Mean arterial pressure for these animals was measured at 58.6 ± 7.4 mm Hg, and oxygen saturation was at 99.5% ± 0.3%. Temperature was also maintained at 98.8°F ± 0.6°F. Post-surgical recovery for these animals also occurred without any significant events.

This model of CCI has been shown to yield a lesion volume of 8% of the hemisphere or 4% of cerebrum. At 5 days post-injury, pigs were euthanized and pericontusional cortical tissue was dissected at ∼2 cm away from the area of the lesion. Contralateral tissue at the equivalent region was also dissected. These samples were then snap-frozen in liquid nitrogen. To generate representative hematoxylin and eosin (H&E)-stained slides, a pig with the same CCI injury was euthanized, and microscopy was performed for histological analysis by a blinded neuropathologist (M.H.).

### RNA extraction and sequencing

Total RNA was extracted from tissue samples by QIAsymphony (Qiagen, Hilden, Germany) automated RNA extraction as previously described.^23^ Briefly, extracted RNA was reverse transcribed to complementary DNA (cDNA) using a reverse transcription kit with RNAse inhibitor (Life Technologies, Grand Island, NY). The Robo-Zero ribosomal RNA (rRNA) Removal Kit was used to remove rRNA (Illumina, San Diego, CA), and the NEBNEXT Ultra RNA Library Prep Kit for Illumina was used to for cDNA library preparation. Sequencing was performed on an Illumina HiSeq instrument by Genewiz, Inc. (Cambridge, MA). Quality of RNA was assessed by checking the bioanalyzer data for each sample, ensuring the 18S and 28S rRNA band's presence.

### Bioinformatics and statistical analysis

Alignment was done using Spliced Transcripts Alignment to a Reference (STAR) to the pig reference genome (SusScorfa 11.1) using default settings for STAR. We then used featurecounts to quantify reads at the gene level using default settings.^24^ Differential expression analysis was done using the *DESeq2* package in R (R Foundation for Statistical Computing, Vienna, Austria) using pair-wise comparison with RNA integrity number, animal identifier, and laterality as independent variables. We used the default Benjamini-Hochberg correction with multiple comparisons, with a corrected *p*-value cutoff of <0.05. Plotting was done using the *ggplot2* package in R. Gene Ontology (GO) analysis was then performed for molecular function, biological process, and reactome analysis.

## Results

### Differentially expressed messenger RNA in pericontusional cortex after traumatic brain injury

**FIG. 1.**
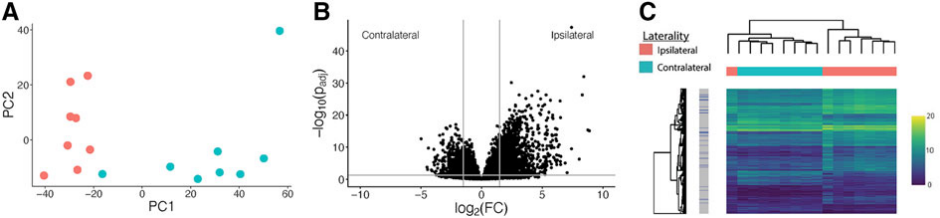
Principal component analysis of contralateral (cyan) and ipsilateral (red) samples. (**A**). Volcano plot showing differentially expressed genes, with negative log_2_(FC) showing increased expression in ipsilateral samples (**B**). Hierarchical clustering map of differentially expressed genes (**C**). Samples are represented by columns, and each gene is represented by a row. FC, fold change; PC1, principal component 1; PC2, principal component 2.

**FIG. 2.**
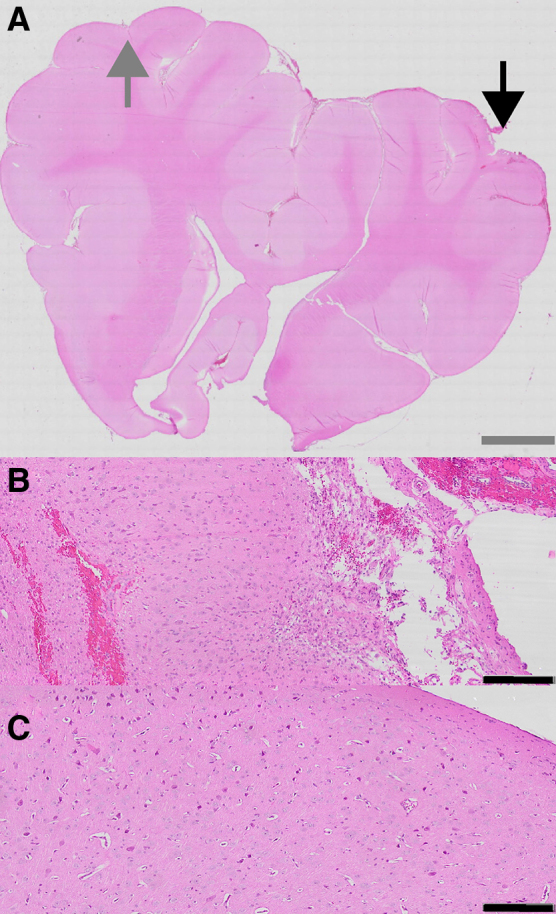
Hematoxylin and eosin staining of the porcine brain after TBI. Low-magnification view of the coronal section of pig brain (**A**), with bar representing 5 mm. Black arrow represents ipsilateral side of injury magnified as shown in (**B**), and gray arrow represents contralateral side of injury magnified as shown in (**C**), with bars representing 200 μm for both slides. TBI, traumatic brain injury.

Among the 16 samples from 8 individual animals, comparison of ipsi- to contralateral side of injury by principal component analysis yielded distinct separation of samples by laterality as shown in Figure 1A, although there was significant variation within the ipsilateral injury cohort. A total of 6642 genes showed significant differences in expression between ipsi- and contralateral sides, with 3520 upregulated and 3122 downregulated on the injured (ipsilateral side; Fig. 1B). Of these, a total of 1993 had > = 3-fold change, with 1396 upregulated and 597 downregulated. As expected from principal component analysis (Fig. 1A), hierarchical clustering showed ipsilateral samples clustering together and contralateral samples clustering together as shown in Figure 1C. There was significant variation in the ipsilateral clustering of genes. To show the severity of injury, the coronal section an H&E-stained pig brain after CCI injury is shown in Figure 2.

### Gene Ontology and pathway analysis

GO analysis yielded protein-protein interaction enrichment *p* values of <10e-16. Differentially expressed genes enriched for biological process, molecular function, and cellular component are shown in Table 1. Molecular function ontologies were enriched for signaling receptor and cytokine receptor activity (219 and 632 genes, respectively). Biological processes were enriched for genes related to immune response (246 genes) regulation of the immune system process (352 genes) and cell-surface receptor signaling pathway (294 genes). Multiple reactome pathways related to the immune system, including the innate immune system (207 genes), neutrophil degranulation (119 genes), and immunoregulatory interactions between lymphoid and non-lymphoid cells (57 genes), were also enriched.

**Table 1.** Top 10 Enriched Gene Ontology and Reactome Terms between Ipsi- and Contralateral Brain Regions Listed by False Detection Rate

	Term	Description	*n*	F.E.	FDR
Molecular function	GO:0022839	Olfactory receptor activity	13	0.12	3.39 × 10^–27^
	GO:0038023	Signaling receptor activity	219	2.17	2.91 × 10^–20^
	GO:0140375	Immune receptor activity	57	6.10	2.33 × 10^–19^
	GO:0004896	Cytokine receptor activity	632	5.60	2.96 × 10^–13^
	GO:0005515	Protein binding	44	1.30	3.08 × 10^–10^
	GO:0019955	Cytokine binding	61	4.22	3.40 × 10^–10^
	GO:0005126	Cytokine receptor binding	32	3.07	1.09 × 10^–9^
	GO:0003723	RNA binding	87	0.37	1.96 × 10^–8^
	GO:0030545	Signaling receptor regulator activity	82	2.19	1.75 × 10^–7^
	GO:0048018	Receptor ligand activity	210	2.22	3.35 × 10^–7^
Biological process	GO:0002376	Immune system process	352	2.81	3.58 × 10^–57^
	GO:0006955	Immune response	246	3.49	2.36 × 10^–52^
	GO:0002682	Regulation of immune system process	275	3.02	1.11 × 10^–48^
	GO:0006952	Defense response	216	3.08	1.96 × 10^–38^
	GO:0002684	Positive regulation of immune system process	185	3.43	9.41 × 10^–38^
	GO:0050776	Regulation of immune response	183	3.46	1.08 × 10^–37^
	GO:0007166	Cell-surface receptor signaling pathway	294	2.31	3.45 × 10^–33^
	GO:0009607	Response to biotic stimulus	211	2.83	4.64 × 10^–33^
	GO:0043207	Response to external biotic stimulus	204	2.85	3.40 × 10^–32^
	GO:0051707	Response to other organism	203	2.84	5.35 × 10^–32^
Reactome	R-SSC-168256	Immune system	327	2.73	3.54 × 10^–51^
	R-SSC-168249	Innate immune system	207	3.14	2.41 × 10^–38^
	R-SSC-6798695	Neutrophil degranulation	119	3.35	3.45 × 10^–23^
	R-SSC-198933	Immunoreg interactions lymphoid/non-lymphoid cell	57	5.94	1.34 × 10^–19^
	R-SSC-1280218	Adaptive immune system	120	2.42	7.04 × 10^–14^
	R-SSC-500792	GPCR ligand binding	70	3.29	5.07 × 10^–13^
	R-SSC-109582	Hemostasis	92	2.63	2.78 × 10^–12^
	R-SSC-202733	Cell-surface interactions at the vascular wall	39	4.53	2.34 × 10^–10^
	R-SSC-373076	Class A/1 (Rhodopsin-like receptors)	57	3.24	2.43 × 10^–10^
	R-SSC-8953854	Metabolism of RNA	5	0.12	1.34 × 10^–9^

F.E., Fisher's exact; FDR, false detection rate; GPCR, G-protein-coupled receptor.

## Discussion

In this report, we identified a large number of differentially expressed genes on the ipsilateral side of a CCI impact compared to the contralateral side. The most notable processes were related to immune system regulation.

Given the anatomical similarities to the human brain based on size and gyrencephalic nature, the pig model has gained more popularity over time compared to the rodent model of TBI. Thus far, transcriptomic analysis showing differentially expressed genes after TBI has not been reported other than in a single study.^11^ In that study, Nikolian and colleagues utilized a combined TBI and hemorrhagic shock model, analyzing the effect of valproic acid 6 h after TBI on perilesional cortex messenger RNA (mRNA) levels. The current study uses a different model of injury with TBI from a CCI and compares gene expression between the ipsi- and contralateral sides of injury. Given the relative lack of literature on the transcriptomic analysis using a large animal model of TBI, we aimed to gain important insights in this aspect of TBI.

Many of the enriched biological process terms included immune-system–related activity, and this is also supported by looking specifically at individual upregulated genes. These immune-system–associated genes include cluster of differentiation (CD) 4 (8-fold change), CD86 (9.1-fold change), interleukin (IL) 17F (20.7-fold change), IL1A (8.1-fold change), and IL23R (48.5-fold change). Inflammatory and immune function gene upregulation is consistent with the known changes of pericontusional tissue at 5 days, further supporting the validity of our analysis.^25, 26^ Additionally, from the current data, we can also gain insight into other major pathological process, such as apoptosis shown by upregulation of CASP10, a gene for major apoptotic molecule caspase-10 (7.0-fold change).

Interestingly, there was upregulation of signal transducer and activator of transcription 6 (STAT6; by 4.4-fold) and a group of known STAT6 targets, suggesting that this may represent a key regulatory molecule in response to TBI. The role of STAT6 in TBI has only been addressed in the setting of concurrent ethanol intoxication, so this represents a potentially novel finding.^27^ Further, IL-4 and IL-13 elevation is known to stimulate STAT6 signaling, and past literature in rodent TBI models showed elevation of these cytokines in the cerebrospinal fluid acutely after TBI.^28^ Protein levels of these two cytokines are also elevated in brain tissue^29^ at 24 h, consistent with the acute time point of the current study. However, there was no increase in IL-4 or IL-13 gene expression in perilesional cortical tissue in our study, making it likely that they may originate outside the brain tissue. Although there was an increase in the IL-4 receptor gene (IL4R) by 6.7-fold, the IL-13 receptor gene (IL13RA2) showed no significant change. Thus, IL-4 signaling may be the main contributor to STAT6 transcription rather than IL-13. Given that STAT6 has also been shown to be involved in chronic inflammatory states, such as allergy, asthma, atopic dermatitis, and eosinophilic esophagitis,^30,31^ this may be a major driver of chronic inflammatory changes in the injured brain post-TBI.

In addition, there was a 3.4-fold elevation of the GATA binding protein 3 gene, a target of STAT6 and a major regulator of T helper 2 (Th2) differentiation. Given that STAT6 plays an important role in differentiating T-helper cells into a Th2 phenotype,^32^ this finding indicates a major drive of the immune system to activate a humoral response at 5 days after TBI. Also, two of the IL-2 receptor subunits (βandγ), which also drive Th2 polarization of T-helper cells, are upregulated (3.4- and 3.9-fold, respectively). In a past study, patients developed a large proportion of activated B cells at 7 days after brain injury.^33^ These B cells have been shown to generate autoantibodies to myelin basic protein, glial fibrillary acidic protein, S100 calcium binding protein B, and glutamate receptors.^34^ Given that these autoantibodies may have an important role in chronic inflammation post-TBI, elevation of STAT6 and associated genes may explain this phenomenon of Th2 differentiation and activation of B cells.

The limitations of the current report include the sizeable number of swine genes that have unknown function. Unlike humans or rodents, there are fewer identified genes in this species, and thus additional implications from the current transcriptomic analysis would require repeated analysis in the future. Moreover, comparison between the ipsi- and contralateral sides of an injured animal may be confounded by compensatory up- or downregulation of genes in the contralateral side. For example, a review of individual differentially expressed genes showed a 4.7-fold decrease in brain-derived neurotrophic factor (BDNF), a major signaling molecule for neural recovery after injury. Instead of downregulation of BDNF in the ipsilateral side, a more sensible explanation is relative upregulation of BDNF on the contralateral side of injury as a compensatory response to TBI. In addition, the techniques used in our analysis do not permit separation by cell type, so some of the inflammatory gene upregulation may be attributable to increased numbers of macrophages or other inflammatory cells in the area of injury.

Also, the current transcriptomic analysis was performed with the assumption of minor to no injury in the contralateral side of impact as shown on H&E staining. This may be true, based on the fact that our CCI was mild to moderate in severity. However, given that severe TBI can often have areas of diffuse injury even remote from the site of impact, a minor degree of injury even at the contralateral side can be a confounding variable.

Transcriptional profiling of pediatric pigs after TBI has been performed at a limited scope previously, looking only at inflammatory cytokine mRNA.^35^ In this past investigation, C-C motif chemokine ligand (CCL) 2 and CCL4 mRNA had significant elevation in cortical lesion compared to the contralateral region. Similarly, IL-1β, tumor necrosis factor alpha, and prostaglandin-endoperoxide synthase 2 showed a clear trend of elevation, but no significant difference from the contralateral side was observed attributable to high variability. Although a direct comparison of immune response to injury between adult and pediatric animals has not been thoroughly assessed in the literature, pediatric TBI likely results in a unique response compared to adults given the role of developmental physiology. In pediatric rodents, both inflammatory cytokines, such as IL-1β,^36^ and anti-inflammatory cytokines, such as IL-10,^37^ are important mediators of neural development. Large fluctuations of inflammatory cytokines throughout early development have been reported,^38^ likely functioning as an integral part of neural development. Especially, female pediatric brain tissue levels of inflammatory cytokines had higher activation compared to males,^39^ further complicating the understanding of immune response as both age and sex dependent.

This investigation was limited to female pediatric pigs, making it unclear whether similar findings would be observed in male pediatric pigs. Given that estrogen and female sex was shown to be neuroprotective in TBI,^40–43^ we investigated the transcriptomic analysis for only female piglets. However, future studies will need to involve both sexes to investigate the degree of sex-associated pathophysiology in piglets.

## Conclusion

This is a first report comparing the transcriptional profiling of injured and uninjured cerebral cortex using a porcine model of TBI. We demonstrate the major transcriptional changes affected in the pericontusional cortex compared to contralateral cortex remote from contusional injury at 5 days post-TBI. The most notable differential expression included various genes in the immune system response. Future transcriptomic studies in a pig model of TBI at various time points and different regions will be helpful in understanding the pathophysiology of TBI.

## Abbreviations Used

BDNF: brain-derived neurotrophic factor
CCI: controlled cortical impact
CCL: C-C motif chemokine ligand
CD: cluster of differentiation
cDNA: complementary DNA
GO: Gene Ontology
H&E: hematoxylin and eosin
IL: interleukin
mRNA: messenger RNA
RNA-seq: RNA sequencing
rRNA: ribosomal RNA
STAR: Spliced Transcripts Alignment to a Reference
STAT6: signal transducer and activator of transcription 6
TBI: traumatic brain injury
Th2: T helper 2
